# Maleimides Designed for Self-Assembly and Reactivity on Graphene

**DOI:** 10.3390/molecules201018856

**Published:** 2015-10-16

**Authors:** Cristina Mattioli, André Gourdon

**Affiliations:** NanoSciences Group, Centre d’Elaboration de Matériaux et d’Etudes Structurales, Centre National de la Recherche Scientifique, CEMES-CNRS, 29 rue Jeanne Marvig, BP 94347, 31055, Toulouse Cedex 4, France; E-Mail: cristinamattioli7@gmail.com

**Keywords:** maleimides, Diels-Alder, graphene

## Abstract

Two new maleimide derivatives have been synthesized, prone to self-assemble and react with graphene as dienophiles. Both compounds bear a long alkyl chain on the carbon-carbon double bond position 3. The maleimide **1** bears a second alkyl chain at the nitrogen, while in compound **2**, three maleimide functionalities are linked to a triethynylbenzene core.

## 1. Introduction

Chemical functionalization of graphene is essential [1] for future practical applications of this attractive material and it has been recently shown that its peculiar electronic structure allows it to react both as a diene or a dienophile in Diels-Alder reaction, thus permitting a large variety of additions in relatively mild conditions [2]. Current work in progress involves the [2 + 2] and [2 + 4] cycloadditions of several maleimides with graphene on silicon carbide surfaces or with graphene flakes supported on silicon dioxide.

In this context, we recently synthesized the derivatives **1** and **2** where the dienophile groups are functionalized maleimides.

Maleimides are a class of substrates arousing interest in many fields of application. Some derivatives possess anticancer, antifungal, herbicidal, or pesticidal properties [3]. The most interesting type of reactivity is shown in Diels-Alder reactions [4,5], as dipolarophiles in 1,3-dipolar cycloadditions [6] or in Michael additions. In particular, the high affinity towards the thiolic functions of the amino acid cysteine, renders the maleimide core as a nice platform for protein recognition [7,8] or for the labelling with fluorescent units [9]. The same maleimide backbone can show fluorescence properties, if bearing phenyl-substituents on the carbon-carbon double bond [10,11]. Other applications see the employment of maleimides as curing agents in epoxy resins [12] or reagents for radicalar polymerizations [13], as well as “active” chemical functionalities in polymers, allowing for further attachment of different moieties through thio-ene [14,15] or Diels-Alder reaction [16].

**Figure 1:**
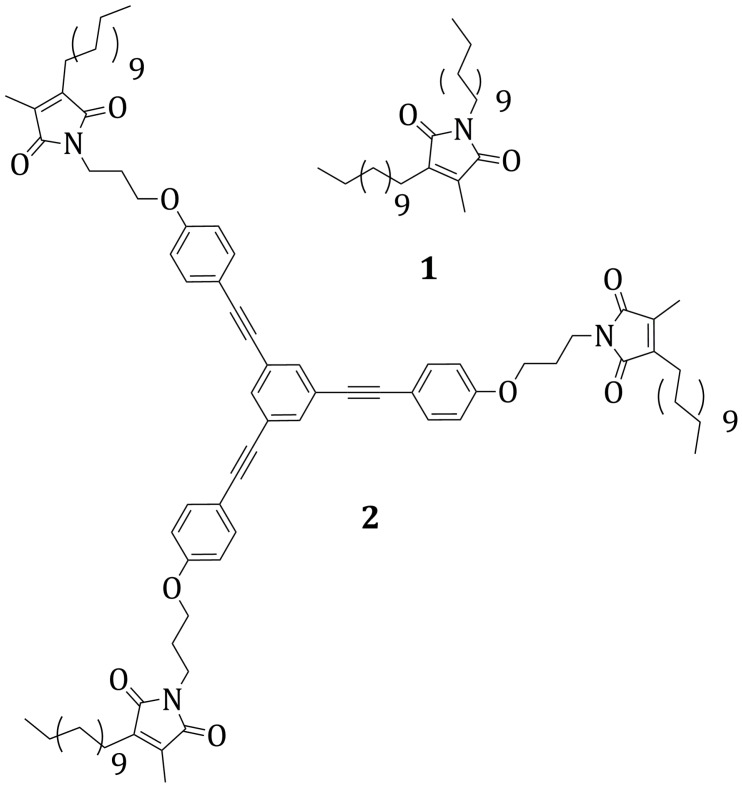
Functionalized maleimides **1** and **2**.

The first maleimide derivative of the series, **1**, bears two alkyl chains –C_12_H_25_ directly connected to the core at positions 1 and 3 (Figure 1). The position 4 is functionalized with a –CH_3_ group, deriving from the particular synthetic strategy that was adopted. The presence of the two long alkyl chains should favour the adsorption of the molecule on the surface, by establishment of CH-π interactions. At the same time, **1** is designed to allow a self-organization of the molecules in linear features on the graphene surface. The second derivative, **2**, presents a slightly more complex structure. The maleimide reactive groups are born by a triethynylbenzene platform. This kind of aromatic core is chosen for two reasons: (1) it favors the molecular adsorption on the surface thanks to π-π interactions; (2) it allows the tuning of reactive sites density on the surface by varying the length of the phenyl-ethynyl spacers. The maleimide groups are functionalized in position 4 by a –CH_3_ group, in position 3 by a –C_12_H_25_ alkyl chain and in position 1 by a –C_3_H_6_ chain, connecting it to the aromatic platform by an ether bond. The C_3_H_6_ chain is adopted in order to guarantee to the maleimide core enough flexibility such as to adapt the correct geometrical conformation upon Diels-Alder reaction. The role of the alkyl chain –C_12_H_25_ is to promote the adsorption on the surface by establishment of CH-π interactions.

## 2. Results and Discussion

### 2.1. Maleimide ***1***

**Scheme 1:**
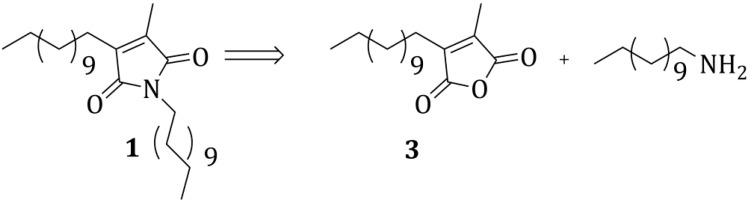
Retrosynthetic analysis for the maleimide derivative **1**.

From a retrosynthetic point of view, **1** can be disconnected to the alkyl-functionalized maleic anhydride **3** and 1-dodecylamine (Scheme 1).

**Scheme 2:**
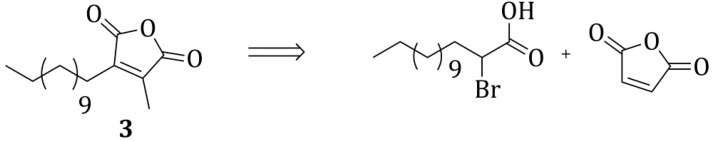
Retrosynthetic analysis for the anhydride derivative **3**.

Different disconnections can be envisaged for the alkyl-functionalized maleic anhydride **3**. The synthesis of this kind of derivatives has largely been studied in the literature, due to their interesting biological activity [17]. The reported disconnection (Scheme 2) appeared the most interesting to us and was already employed by Argade *et al.* for the synthesis of “Chaetomellic Anhydride A” [18].

**Scheme 3:**
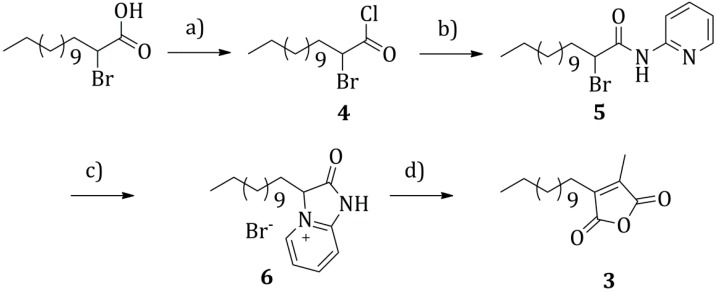
Synthesis of the alkyl-functionalized maleic anhydride **3**. Conditions: (**a**) oxalyl chloride (1.6 eq.), DMF cat., dry toluene, 20 °C, 2 h; (**b**) 2-aminopyridine (1 eq.), triethylamine (1 eq.), dry diethyl ether, 20 °C, 3 h; (**c**) *tert*-butanol, 82 °C, 18 h; (**d**) maleic anhydride (1 eq.), sodium acetate (1 eq.), acetic acid, 120 °C, 5 h. Yield over the four steps: 27%.

The strategy leads to disconnect the anhydride **3** directly to maleic anhydride and 2-bromo-tetradecanoic-acid. All the synthetic steps could be performed in a one-pot fashion (Scheme 3).

**Scheme 4:**
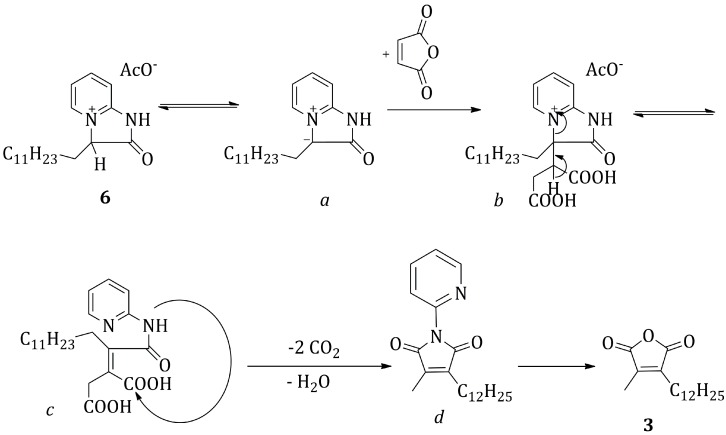
Mechanism of formation of the functionalized maleic anhydride **3** from compound **6**.

In a first step (Scheme 3a), 2-bromo-tetradecanoic-acid was activated to an acyl chloride, by employing an excess of oxalyl chloride, in dry toluene at room temperature for 2 h, under an argon atmosphere. The product was obtained as a yellow oil after evaporation of the solvent and was employed without any purification in the following step. The second step (Scheme 3b) consisted in a nucleophilic acyl substitution by 2-aminopyridine. The reaction was done in dry diethyl ether at room temperature for 3 h, employing triethylamine as a base. Product **5** was obtained as a yellow solid after solvent evaporation under reduced pressure and employed without any purification in the following step. In the third step (Scheme 3c), the derivative **5** was cyclized to product **6** by stirring under reflux of *tert*-butanol for 18 h. **6**, obtained as a dark thick oil after solvent evaporation, was employed for the following reaction without any purification. The last step (Scheme 3d) consisted in reacting **6** with maleic anhydride, in presence of one equivalent of sodium acetate, in refluxing acetic acid for 5 h. The proposed mechanism (Scheme 4) for this last step is similar to the one described by Bauman *et al.* for the similar synthesis of dimethylmaleic anhydride [19]. The proton in alpha to the carbonyl group of **6** is made particularly acidic by the presence of the quaternary nitrogen substituent. In the employed conditions, it is possible to assume that the protonated and deprotonated forms are in equilibrium: the so formed carbanion *a* can act as a Michael donor and attack maleic anhydride in position 3, to give an adduct *b* that after a sequence of reactions (β-elimination, ring opening and decarboxylation) yields the 1-pyridine-3-dodecyl-4-methyl functionalized maleimide *d*. Hydrolysis in the acidic environment followed by cyclization leads to the desired 3-dodecyl-4-methyl maleic anhydride **3**.

The product **3** could be isolated by silica gel column chromatography (eluent petroleum ether/diethyl ether 9:1) as a yellow oil, with a yield over four steps of 27%.

**Scheme 5:**
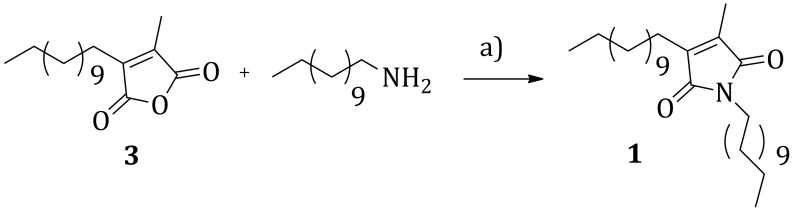
Synthesis of the maleimide derivative **1**: (**a**) 1-dodecylamine, ZnBr_2_, HMDS, dry toluene, 80 °C, 19 h, 89%.

The alkyl functionalized maleic anhydride **3** was reacted with 1-dodecylamine to yield the maleimide **1** (Scheme 5).

The employed conditions were adapted to our case from those suggested by Reddy *et al.* for similar compounds. The anhydride ring was opened by the carbonyl addition of 1-dodecylamine, in toluene at room temperature. After taking the mixture to 80 °C, the Lewis acid zinc dibromide was added, followed by hexamethyldisilazane and the mixture was then refluxed for 19 h, to afford the cyclized maleimide with 89% yield. Although the mechanism of the amic acid cyclization is not yet clearly defined, it is assumed to consist in a Lewis acid/ HMDS promoted sylilation of an intermediate amic acid to a labile trimethylsilyl ester, followed by subsequent thermal deoxysilylation [20]. It is interesting to notice that the cyclization reaction by more “standard” conditions [17] (*i.e.*, refluxing in acetic anhydride in the presence of sodium acetate), was unsuccessful in this particular case and only the starting reagent was recovered. The product was obtained as yellow oil. The 2D ^1^H-NMR characterizations confirmed the disubstitution by the two –C_12_H_25_ alkyl chains with in particular the chemical shifts N-CH_2_ at 3.44 ppm and C=C–CH_2_ at 2.35 ppm.

### 2.2. Maleimide ***2***

**Scheme 6:**
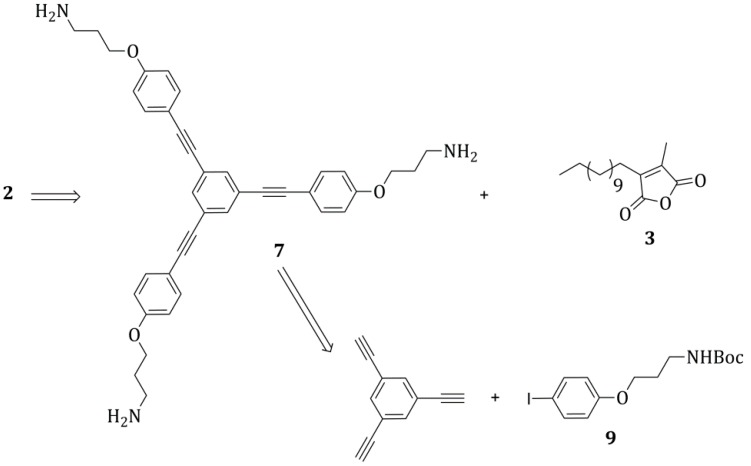
Retrosynthetic analysis for the maleimide **2**.

Among the different retrosynthetic paths that can be envisaged, we choose to adopt the one based on the formation of the maleimide bond at the end of the synthesis, by reacting the amine-nitrogen atoms of the precursor **7** with the formerly prepared functionalized maleic anhydride **3** (Scheme 6). The precursor **7** could be obtained by deprotection of the product obtained by coupling 1,3,5-triethynylbenzene and the compound **9**.

#### 2.2.1. Synthesis of **9**

**Scheme 7:**
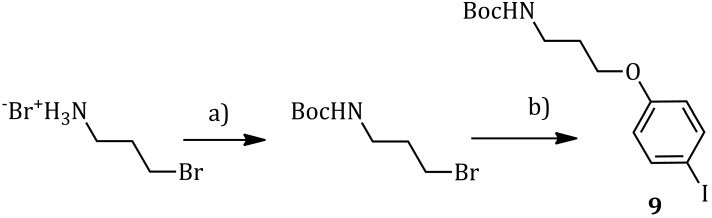
Synthesis of the precursor **9**: (**a**) NaOH, Boc_2_O, H_2_O/THF, 0 °C–22 °C, 5 h, 90%; (**b**) *p*-iodophenol, Cs_2_CO_3_, NaI, dry CH_3_CN, 65 °C, 5 h, 83%.

*Tert*-butyl 3-bromopropylcarbamate was synthesized by a literature procedure [21]. The second step consisted in the *O*-alkylation of *p*-iodophenol with *tert*-butyl 3-bromopropylcarbamate. *p*-Iodophenol was deprotonated by cesium carbonate in acetonitrile at 65 °C; the as-generated nucleophile was then alkylated in a S_N_2 reaction, promoted by sodium iodide [22]. Product **9** was obtained with an 83% yield as a yellow solid after column chromatography on silica gel (Scheme 7).

#### 2.2.2. Synthesis of **7**

**Scheme 8:**
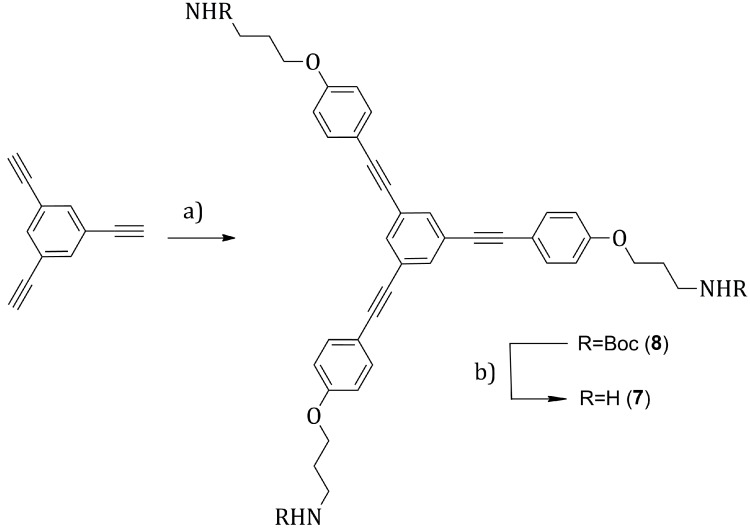
Synthesis of the precursor **7**: (**a**) **9**, Pd(PPh_3_)_2_Cl_2_ (6%), CuI (2.3%), dry THF/Et_2_NH, 45 °C, 4 h, 48%; (**b**) µW (230 W), DMF, 180 °C, 10 min, 100%.

Compound **8** was obtained by Sonogashira cross coupling between 1,3,5-triethynylbenzene [23,24] and the precursor **9** (Scheme 8a). The reagents were stirred in dry tetrahydrofuran/diethylamine, in the presence of bis(triphenylphosphine) palladium(II) dichloride (2% per alkyne) and copper iodide (0.8% per alkyne) as catalyst. The reaction temperature was optimized at 45 °C and the reaction time to 4 h. The moderate warming allowed to speed up the reaction, in particular the palladium C-I insertion step, unfavored by the presence of the electron donor substituent in *para* to the iodine on **9**. Under these conditions, the product **8** was obtained after a simple column chromatography (cyclohexane/ethyl acetate 6:4) with a 48% yield as a light yellow solid. Alternative conditions, such as: tetrahydrofuran/triethylamine as solvents, copper iodide (2.7% per alkyne) and tetrakis(triphenylphosphine)palladium(0) (1.3% per alkyne) as catalyst, at room temperature (22 °C) for 18 h, allowed as well to obtain **8**, but the yields were slightly lower and less reproducible (ranging from 24% to 48%) and the purification more difficult. In the following step (Scheme 8b), the *tert*-butyloxycarbonyl (Boc) protecting group was removed to generate the free-amine **7***.*

**Scheme 9:**
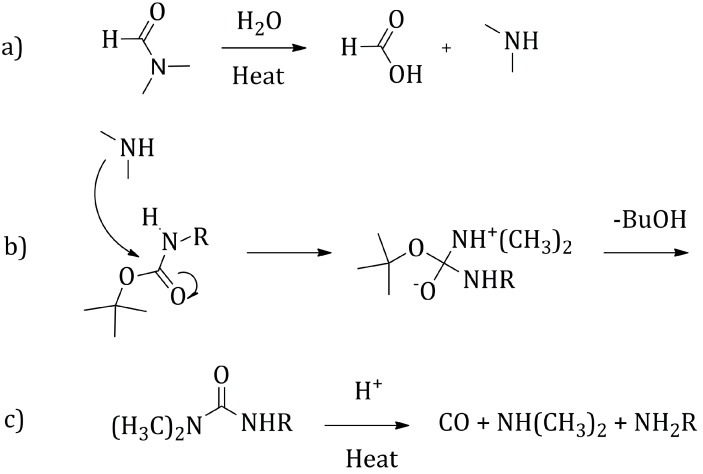
Proposed mechanism for the Boc thermal decomposition.

A solution of **8** in *N*,*N-*dimethylformamide (0.04 M) was irradiated with microwaves at a power of 230 Watts, adjusting the microwave reactor parameters such as to keep the reaction temperature at 180 °C for 10 min (Scheme 8b) [25,26]. In these conditions, the Boc decomposition was quite rapid and **7** could be recovered quantitatively as a brown thick oil, showing only a partial solubility in dimethylsulfoxide. Due to the high polar character of the molecule, related to the presence of the free amines, it was not possible to carry on a chromatographic purification, but the product was just treated with petroleum ether in order to remove some non-polar impurities. Likely, the deprotection mechanism in the presence of *N*,*N-*dimethylformamide, could be related to the solvent decomposition in formic acid and dimethylamine, at high temperatures (Scheme 9) [27]. The as-generated dimethylamine is the nucleophilic species attacking the Boc carbonyl, determining the formation of a urea group, that then thermally decomposes to finally yield the free amines.

We tested also a standard deprotection procedure in acidic medium. **8** was dissolved in a solution 4 M of hydrochloric acid in tetrahydrofuran and the reaction run at 0 °C for 10 min then at 22 °C for 30 min. The product could be recovered after basic work up, by extraction with ethyl acetate with yields going up to 75%. However, the extraction procedure suffered from lack of reproducibility and many difficulties were encountered in the recovery of **7**, probably due to its insolubility in ethyl acetate. Another problem that we had to deal with was the cleavage of the phenolic-ether bond that can be promoted in these conditions [28], leading to the recovery of the only alkyl chain moiety.

#### 2.2.3. Synthesis of **2**

**Scheme 10:**
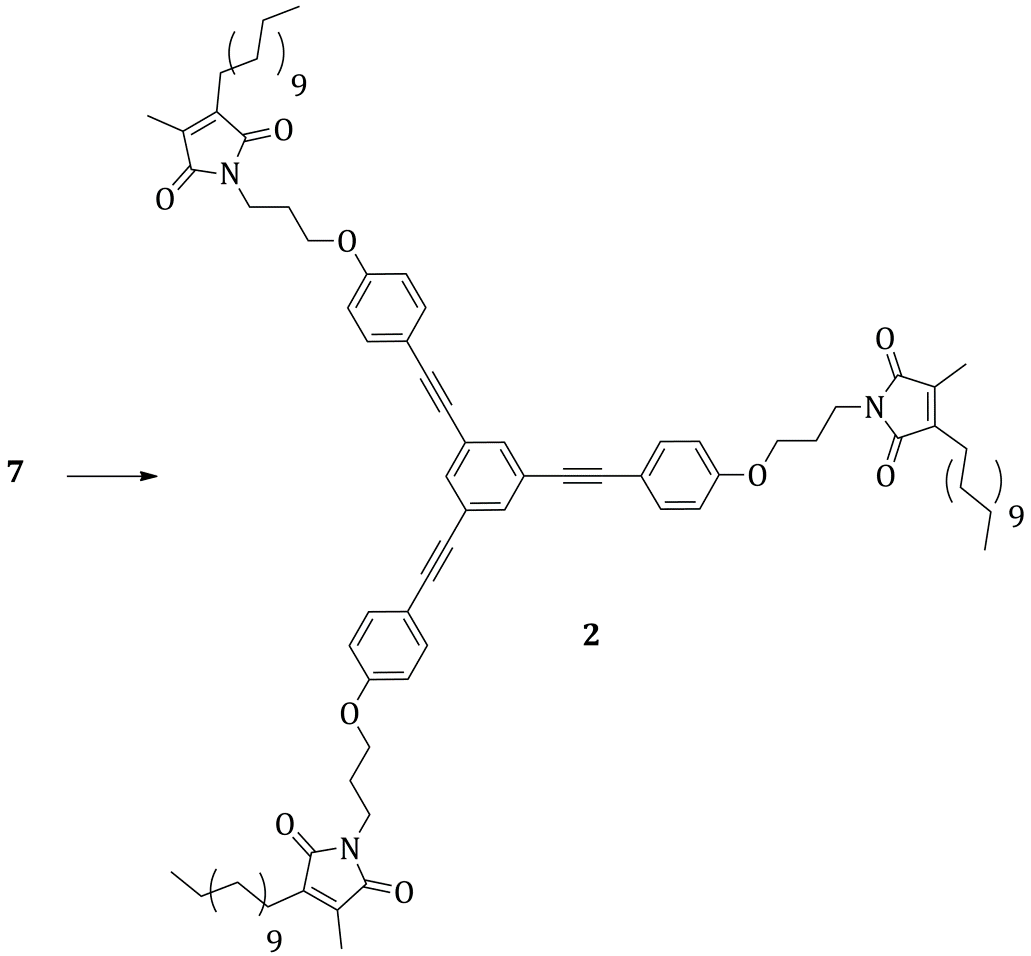
Synthesis of the tri-maleimide **2**: (i) **3**, dry toluene, 111 °C, 16 h; (ii) ZnBr_2_, HMDS, 80 °C, 5 h, then 22 °C, 11 h, 20%.

The tri-amine **7** was condensed with the maleic anhydride **3** to yield the tri-maleimide **2** (Scheme 10). The reaction was carried out analogously to the synthesis of the functionalized maleimide **1**.

The anhydride **3** cycle was at first opened by reacting it in an acyl nucleophilic substitution with the amine nitrogens of **7**, in toluene. Due to the insolubility of **7** in toluene, a solution of the anhydride **3** in toluene was added to the flask containing the thick oil **7** and the mixture was warmed at 111 °C for 16 h. With this procedure, **7** could be brought in solution as its derived carbamic acid, which was then cyclized to **2** by employing the system ZnBr_2_/HMDS described above. The compound **2** could then be recovered by two column chromatography purifications on silica gel (hexane/ethyl acetate 6:4; then hexane/diethyl ether 95:5, followed by diethyl ether 100%) with a 20% yield. The identity and purity of the product were confirmed by ^1^H-NMR spectroscopy and high-resolution mass spectrometry. The global yield over the five steps from 3-bromopropylamine hydrobromide was 5%.

In summary, we have developed the syntheses of two maleimide derivatives designed for self-assembly on graphene and Diels-Alder cycloadditions with this material in order to carry out controlled functionalization in mild conditions. The strategy that we adopted consisted in the synthesis of the alkyl-functionalized anhydride precursor and its successive reaction with the amine precursor for the generation of the maleimide core. Current work involves transfers on graphene surfaces in ultra-high vacuum, imaging of self-assembled monolayers by Scanning Tunneling Microscopy and tip-induced controlled activation of cycloadditions.

## 3. Experimental Section

### General Information

1,3,5-Tris[(trimethylsilyl)ethynyl)benzene and 1,3,5-tri-ethynylbenzene were synthesized by literature procedures from the references [23,24]. *Tert*-butyl 3-bromopropylcarbamate was synthesized by a literature procedure [17]. Solvents were dried over molecular sieves prior to use. Dry DMF was from Acros Organics (Morris Plains, NJ, USA) or Sigma Aldrich (St. Louis, MO, USA). THF was distilled on sodium/benzophenone. Flash column chromatography was performed by using silica gel (60 Å pore size, 40–63 µm Merck, Kenilworth, NJ, USA). The reactions were monitored by thin layer chromatography (TLC) on silica gel-coated plates (Merck 60 F_254_). Detection was performed by using UV light and by charring the plate at *ca.* 200 °C, after dipping it in an ethanolic solution of potassium permanganate. The yields refer to chromatographically and spectroscopically (^1^H-NMR and ^13^C-NMR) homogeneous materials, unless otherwise stated. The NMR spectroscopic data were recorded with Bruker Avance 300/400/500 MHz (Bruker, Fremont, CA, USA) instruments and were calibrated by using the residual undeuterated solvent as an internal reference (CDCl_3_ at δ_H_ = 7.26 ppm, δ_C_ = 77.16 ppm, CD_2_Cl_2_ at δ_H_ = 5.33 ppm, δ_C_ = 53.84 ppm). Chemical shifts are reported in parts per million (ppm) on the δ scale and coupling constants (J) are in Hertz (Hz). The abbreviations used to describe the multiplicities are s = singlet, bs = broad singlet, d = doublet, t = triplet, dd = doublet of doublets, q = quintuplet, m = multiplet. Mass spectra were recorded at the Service Commune de Spectrometrie de Masse of University Paul Sabatier (Toulouse 3), Toulouse (France). Elemental analyses were done by the Service d’Analyse de l’ICSN (Paris, France). Microwave heating was carried out in closed vials with a CEM-Discovery monomode microwave apparatus under the specified conditions (power, temperature, time).

*2-Bromo-tetradecanoic-chloride* (**4**): In an oven dried three neck flask, under an atmosphere of argon, 13 mL (3.31 g, 26.08 mmol) of a 2 M solution of oxalyl chloride in dichloromethane were added to a solution of 5 g (16.3 mmol) of 2-bromotetradecanoic acid in 80 mL of dry toluene, followed by a drop of dimethylformamide. The mixture was stirred for 2 h at room temperature, and then the solvent was removed under reduced pressure to give **4** as a yellow oil. The product was used for the following reaction without any further purification.

*2-Bromo-N-(pyridin-2-yl) tetradecanamide* (**5**): To a solution of 2-aminopyridine (1.53 g, 16.3 mmol) and dry triethylamine (2.27 g, 16.3 mmol) in dry ether (100 mL) at room temperature under argon, was added the chloride **4** (5.3 g, 16.3 mmol) in dry ether (30 mL) in a dropwise fashion over a period of 20 min under vigorous stirring. The reaction mixture was further stirred for 3 h at R.T. (20 °C) and the solvent removed under vacuum. The product (a yellow solid) was used for the following reaction without any further purification.

*3-Dodecyl-2-oxo-2,3-dihydro-1H-imidazo [1,2-a] pyridin-4-ium Bromide* (**6**): The total residue (**5**) was dissolved in 100 mL of *tert*-butanol and warmed at reflux (82 °C) for 18 h. The solvent was removed under reduced pressure, giving an orange oil that was used for the following step without any purification.

*3-Dodecyl-4-methylfuran-2,5-dione* (**3**): The residue **6** was reacted with maleic anhydride (1.60 g, 16.3 mmol) in the presence of sodium acetate (1.34 g, 16.3 mmol) in 98% acetic acid (100 mL), under reflux (120 °C) for 5 h under constant stirring. Acetic acid was then removed under vacuum and the residue dissolved in ether. The ether layer was washed with water (2 × 100 mL), brine (100 mL) and dried over magnesium sulphate. The product **3** was obtained as a yellow oil after column chromatography on silica gel (eluent petroleum ether/diethyl ether 9:1, R*_f_* = 0.53), with a 27% yield (over 4 steps). ^1^H-NMR (500 MHz, CDCl_3_): δ (ppm) 2.45 (t, *J* = 7.5 Hz, 2H, C=C–CH_2_), 2.07 (s, 3H, C=C–CH_3_), 1.5–1.6 (m, 2H, CH_2_–CH_2_ ), 1.2–1.3 (m, 18H, CH_2_ (chain)), 0.87 (t, *J* = 7 Hz, 3H, CH_3_ terminal).^13^C-NMR (125 MHz, CDCl_3_): δ (ppm) 164, 166.2, 145.1, 140.8, 32.2, 30.0, 29.9, 29.8, 29.7, 29.5, 24.8, 23.0, 14.5, 9.9. MS (DCI/NH_3_^+^): *m*/*z* = 298 [M + NH_4_]^+^. Elemental Analysis: %C 72.70 %H 9.70 %O 17.60 (found for C_17_H_28_O_3_) *vs.* %C 72.82 %H 10.06 %O 17.12 (calculated for C_17_H_28_O_3_).

*1,3-Didodecyl-4-methyl-1H-pyrrole-2,5-dione* (**1**): To a stirred solution of anhydride **3** (0.770 g, 2.7 mmol) in dry toluene (20 mL) at R.T. (22 °C), a solution of 1-dodecylamine (0.5 g, 2.7 mmol) in dry toluene (10 mL) was added dropwise. The resulting suspension was stirred for one hour, and then zinc bromide (0.61 g, 2.7 mmol) was added in one portion. While the resulting reaction mixture was heated at 80 °C, a solution of hexamethyldisilazane (0.66 g, 0.86 mL, 4.1 mmol) in 10 mL of dry toluene was added drop by drop, and then the mixture was refluxed for 19 h. The reaction mixture was cooled to RT and poured into 0.5 N HCl (20 mL). The aqueous phase was extracted with ethyl acetate (3 × 30 mL). The combined organic extracts were dried over anhydrous magnesium sulphate. The solution was concentrated under reduced pressure and the residue purified by silica gel chromatography (Petroleum ether/diethyl ether 95:5), to afford the product as a yellow oil with a 89% yield.^1^H-NMR (500 MHz, CDCl_3_): δ (ppm) 3.44 (t, *J* = 7.5 Hz, 2H, N–CH_2_), 2.35 (t, *J* = 7.5 Hz, 2H, C=C–CH_2_), 1.94 (s, 3H, C=C–CH_3_), 1.4–1.5 (m, 4H, maleimide–CH_2_–CH_2_), 1.2–1.3 (m, 36H, CH_2_ (chain)), 0.87 (t, *J* = 6.5 Hz, 6H, CH_3_ terminal). ^13^C-NMR (125 MHz, CDCl_3_): δ (ppm) 177.8, 172.4, 141.3, 137.0, 36.3, 32.3, 30.0, 29.9, 29.8, 29.7, 29.6, 29.5, 29.0, 28.6, 27.1, 24.0, 23.0, 14.5, 9.0. MS (DCI/NH_3_^+^): *m*/*z* = 465 [M + NH_4_]^+^. Elemental Analysis: %C 77.58 %H 11.32 %N 3.13 %O 7.97 (found for C_29_H_53_NO_2_) *vs.* %C 77.79 %H 11.93 %N 3.13 %O 7.15 (calculated for C_29_H_53_NO_2_).

*Tert-butyl 3-(4-iodophenoxy)propylcarbamate* (**9**): To a solution of *p*-iodophenol (2 g, 9.1 mmol) and *tert*-butyl-3-bromopropylcarbamate (2.38 g, 10 mmol) in dry acetonitrile (100 mL), were added Cs_2_CO_3_ (4.45 g, 13.65 mmol) and NaI (0.34 g, 2.28 mmol) and the mixture was let to react at 65 °C for 5 h. The inorganic salts were filtered away and the solvent evaporated under reduced pressure. The product, a light yellow solid, was purified by column chromatography on silica gel (pentane/diethyl ether 7:3, R*_f_* = 0.5) with a 83% yield. Melting point: 78–80 °C. ^1^H-NMR (300 MHz, CDCl_3_): δ (ppm) 7.53 (dt, 2H, *J*_1_ = 9 Hz, *J*_2_ = 2.1 Hz, Ar–H (orto to O)), 49 (dt, 2H, *J*_1_ = 9 Hz, *J*_2_ = 2.1 Hz, Ar–H (ortho to I)), 4.72 (bs, 1H, –NH–), 3.97 (t, 2H, *J* = 6 Hz, –O–CH_2_–), 3.31 (q, 2H, *J* = 6 Hz, –NH–CH_2_–), 1.96 (q, 2H, *J* = 6.3 Hz, CH_2_–CH_2_–CH_2_), 1.43 (s, 9H, –C(CH_3_)_3_).^13^C-NMR (75 MHz, CDCl_3_): δ (ppm) 158.9, 156.3, 138.5, 117.2, 83.1, 66.2, 38.2, 29.8, 28.7. MS (DCI/NH_3_^+^): 338.9 [M − C(CH_3_)_3_ + H]^+^, 378.0 [M + H]^+^. HR-MS (DCI CH_4_^+^/TOF): *m*/*z* 377.0488 for C_14_H_20_INO_3_ (calculated 377.0488) (13%); 278.0049 found for C_9_H_13_INO^+^, (calculated 278.0036) (100%).

*Tert-butyl 3,3',3''-(4,4',4''-(benzene-1,3,5-triyltris(ethyne-2,1-diyl))tris(benzene-4,1-diyl))tris(oxy)tris (propane-3,1-diyl) tricarbamate* (**8**): *procedure a)*: **9** (1.57 g, 4.2 mmol) and 1,3,5-triethynylbenzene (0.20 g, 1.3 mmol) were dissolved in distilled tetrahydrofuran (20 mL) and dry diethylamine (13 mL). Argon was bubbled for 15 min then copper iodide (0.006 g, 0.03 mmol) and bis(triphenylphosphine)palladium(II) dichloride (0.06 g, 0.08 mmol) were added and the mixture was let to react at 45 °C for 4 h. The mixture was filtered on a silica pad (washing with ethyl acetate) and the solvent evaporated under reduced pressure. The product (a slightly yellow solid) was obtained pure by column chromatography on silica gel with cyclohexane/ethyl acetate 6:4 as eluent. (R*_f_* = 0.4 in cyclohexane/ethyl acetate 6:4). The yield was 48%. Melting point: 104–106 °C. ^1^H-NMR (300 MHz, CD_2_Cl_2_): δ (ppm) 7.59 (s, 3H, Ar–H), 7.49 (d, 6H, *J* = 8.7 Hz, Ar–H), 6.91 (d, 6H, *J* = 8.7 Hz, Ar–H), 4.82 (bs, 2.5H, –NHCO), 4.05 (t, 6H, *J* = 6.3 Hz, CH_2_), 3.31 (q, 6H, *J* = 4 Hz, CH_2_), 1.98 (q, 6H, *J* = 4 Hz, CH_2_), 1.44 (s, 27H, –CO(CH_3_)_3_).^13^C-NMR (75 MHz, CD_2_Cl_2_): δ (ppm) 160.1 (–C=O), 154 (Ar–C), 134.1 (Ar–C), 134.0 (Ar–C), 125.1 (Ar–C), 115.6 (Ar–C), 115.4 (Ar–C), 91.2 (C–OR or C≡C), 87.3 (C≡C), 79.6 (C≡C), 65 (CH_2_), 38.6 (CH_2_), 30.4 (CH_2_), 28.9 (–C(CH_3_)_3_). HR-MS (ESI^+^/TOF): 920.4 [M + Na]^+^ (calculated 920.4), 933 [M + K]^+^ (calculated 933).

Procedure (b): **9** (1.92 g, 5.1 mmol) and 1,3,5-tri-ethynylbenzene (0.24 g, 1.6 mmol) were dissolved in distilled tetrahydrofuran (25 mL) and triethylamine (7 mL). Argon was bubbled for 10 min then CuI (0.024 g, 0.128 mmol) and Pd(PPh_3_)_4_ (0.074 g, 0.064 mmol) were added and the mixture was let to react at room temperature (22 °C) for 18 h. The mixture was filtered on celite (washing with tetrahydrofuran) and the solvent evaporated under reduced pressure. The product (a yellow solid) was obtained pure by two column chromatographies on silica gel: (1) pentane/diethyl ether 6:4, then ethyl acetate; (2) pentane/ethyl acetate 6:4. (R*_f_* = 0.3 in pentane/ethyl acetate 7:3). The yields were ranging between 24% and 48%.

*3,3',3''-(4,4',4''-(Benzene-1,3,5-triyltris(ethyne-2,1-diyl)) tris (benzene-4,1-diyl))tris(oxy)tripropan-1-amine* (**7**): *deprotection by microwave irradiation*: In a microwave reactor equipped with a magnetic stirrer, **8** (0.105 g, 0.12 mmol) was dissolved in 3 mL of dry dimethylformamide. The solution was warmed by microwave irradiation setting the parameters as to keep the temperature at 180 °C for 10 min (PW 230 Watts). TLC analysis of the brown suspension revealed the disappearance of the reagent **8** and the appearance of a new spot characteristic of the desired product at R*_f_* = 0 (on SiO_2_, with hexane/ethyl acetate 6:4 as eluent). After evaporation of the solvent under reduced pressure, a thick brown oil was obtained. The oil was washed twice with petroleum ether and used without any purification for the next step. The yield was quantitative.

Note: The product is only soluble in DMSO and does not elute on TLC stationary phases (SiO_2_, Al_2_O_3_ neutral), so it was directly used without any purification for the next step.

Deprotection by acidic treatment: **8** (0.180 g, 0.2 mmol) was dissolved in 10 mL of a solution 4 M of HCl in THF at 0 °C. The mixture was stirred at 0 °C for 10 min, then at room temperature (25 °C) for 30 min. The solvent was evaporated under vacuum, and then 10 mL of water were added, causing the precipitation of a white solid. The pH was basified to 14 by adding a solution of NaOH 0.01 M. The water phase was extracted with ethyl acetate (3 × 30 mL). The combined organic phases were dried on MgSO_4_ and evaporated. Since NMR analysis revealed that the reagent was not completely deprotected, the reaction was restarted in the same conditions and let to stir at room temperature for 6 h. The product was obtained with a 75% yield and used for the following reaction without further purification. (R*_f_* = 0 both on SiO_2_ and Al_2_O_3_ neutral, with hexane/ethyl acetate 6:4 as eluent). ^1^H-NMR (300 MHz, CD_2_Cl_2_): δ (ppm) 7.64 (s, 3H, Ar–H), 7.52 (d, 6H, *J* = 8.7 Hz, Ar–H), 7.01 (d, 6H, *J* = 8.7 Hz, Ar–H), 4.0–4.1 (m, 6H, CH_2_), 2.7–2.9 (m, 6H, CH_2_), 1.8–1.9 (m, 6H, CH_2_).

*1,1',1''-(3,3',3''-(4,4',4''-(Benzene-1,3,5-triyltris(ethyne-2,1-diyl))tris(benzene-4,1-diyl))tris(oxy)tris(propane-3,1-diyl))tris(3-dodecyl-4-methyl-1H-pyrrole-2,5-dione)* (**2**): To a round bottom flask containing the tri-amine **8** (0.110 g, 0.2 mmol), a solution of the alkylated maleic anhydride **4** (0.21 g, 0.7 mmol) in 8 mL of dry toluene was added. The mixture was let to react at 111 °C for 16 h, giving a brown solution. Toluene was evaporated under reduced pressure. The obtained brown oil was redissolved in fresh dry toluene (6 mL) and transferred in a three neck flask, equipped with a dropping funnel and argon entry. Zinc bromide (0.13 g, 0.6 mmol) was added in one portion at room temperature, then the suspension was warmed at 80 °C. A solution of hexamethyldisilazane (0.15 g, 0.19 mL, 0.9 mmol) in 2 mL of dry toluene was added drop by drop. The mixture was let to react at 80 °C for 5 h then at room temperature (20–22 °C) for 11 h. The reaction was quenched by adding 25 mL of water. Ethyl acetate was added (30 mL), forming an emulsion. The emulsion was extracted with 2 × 30 mL of ethyl acetate, dried on anhydrous MgSO_4_ and evaporated under reduced pressure. The product was obtained as a yellow oil by two column chromatography purifications on silica gel: a first chromatography (eluent hexane/ethyl acetate 6:4) allowed to recover **2** together with an impurity (fractions R_f_ = 0.8 to 0.9). This mixture was further chromatographed (hexane/diethyl ether 95:5, then diethyl ether 100%) to obtain pure **2** in 20% yield. ^1^H-NMR (500 MHz, CDCl_3_): δ (ppm) 7.59 (s, 3H, Ar–H), 7.48 (d, 6H, *J* = 8.7 Hz, Ar–H *ortho* to CC), 6.87 (d, 6H, *J* = 8.7 Hz, Ar–H *ortho* to O), 4.00 (t, 6H, *J* = 6 Hz, N–CH_2_), 3.68 (t, 6H, *J* = 6.5 Hz, O–CH_2_), 2.36 (t, 6H, 7 Hz, C=C–CH_2_), 2.07 (m, 6H, CH_2_CH_2_ CH_2_), 1.95 (s, 9H, C=C–CH_3_), 1.4–1.5 (m, 6H, CH_2_CH_2_), 1.2–1.3 (m, 54H, CH_2_, chain), 0.88 (t, 9H, *J* = 7 Hz, CH_3_ terminal). ^13^C-NMR (125 MHz, CDCl_3_): δ (ppm) 173.0, 172.7, 160.0, 141.9, 137.7, 134.0, 133.9, 125.1, 115.6, 115.3, 91.2, 87.3, 64, 35.8, 32.7, 30.1–30.4, 29.0, 28.9, 24.3, 23.4, 14.7, 9.2. HR-MS (MALDI, DCTB/TOF): *m*/*z* 1383.8790 found for C_90_H_117_N_3_O_9_, 1383.8790 calculated for C_90_H_117_N_3_O_9_.

Copies of ^1^H- and ^13^C-NMR spectra of products **1**, **2**, **3**, **8** and **9** can be found in the supplementary materials.

## Supplementary Materials

Supplementary data associated with this article are available at: http://www.mdpi.com/1420-3049/20/10/18856/s1.
