# Supplementary material for: Maleimides Designed for Self-Assembly and Reactivity on Graphene
Source: Molecules. 2015 Oct 16;20(10):18856–69. doi: 10.3390/molecules201018856 (PMC6331833; doi:10.3390/molecules201018856)
Supplement: Supplementary file 1 [file molecules-20-18856-s001.pdf]

Supplementary Materials

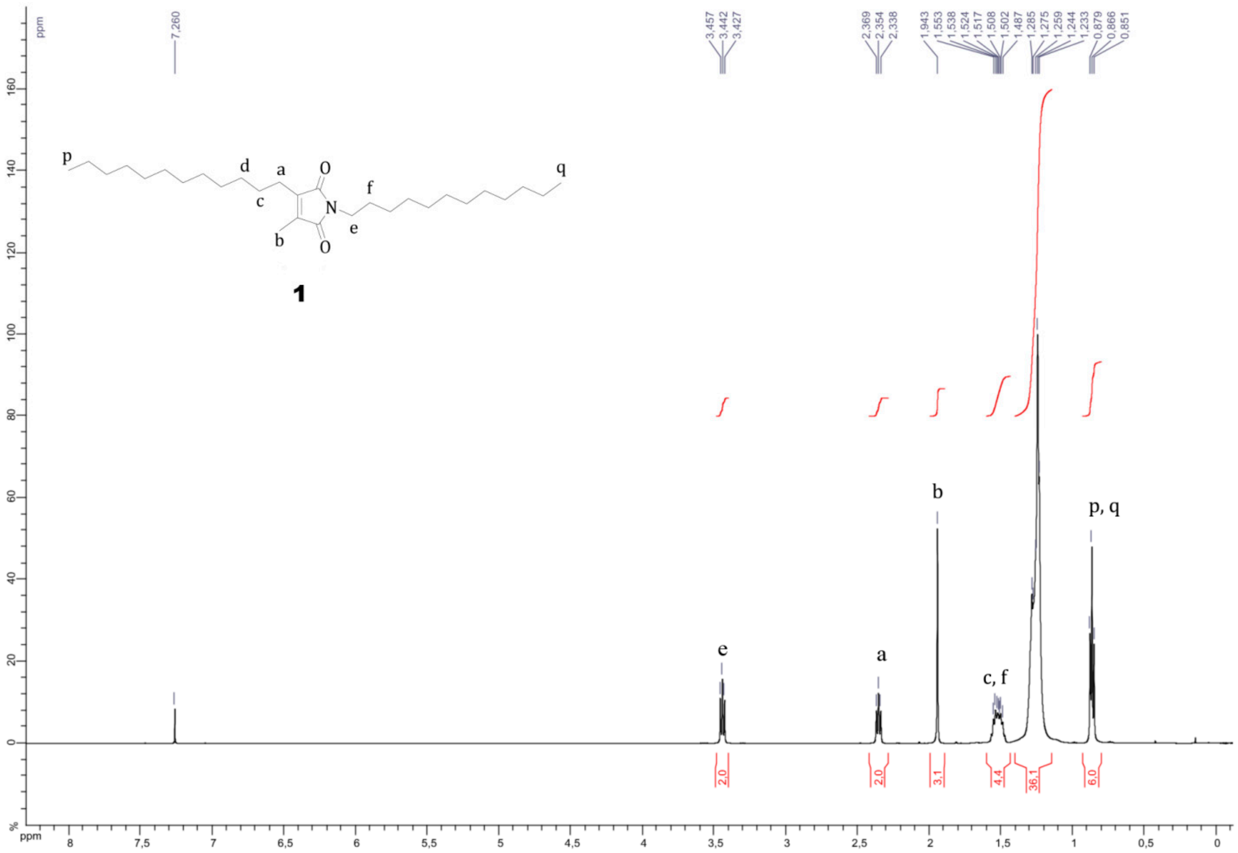

Figure S1. ^1H-NMR of compound **1**.

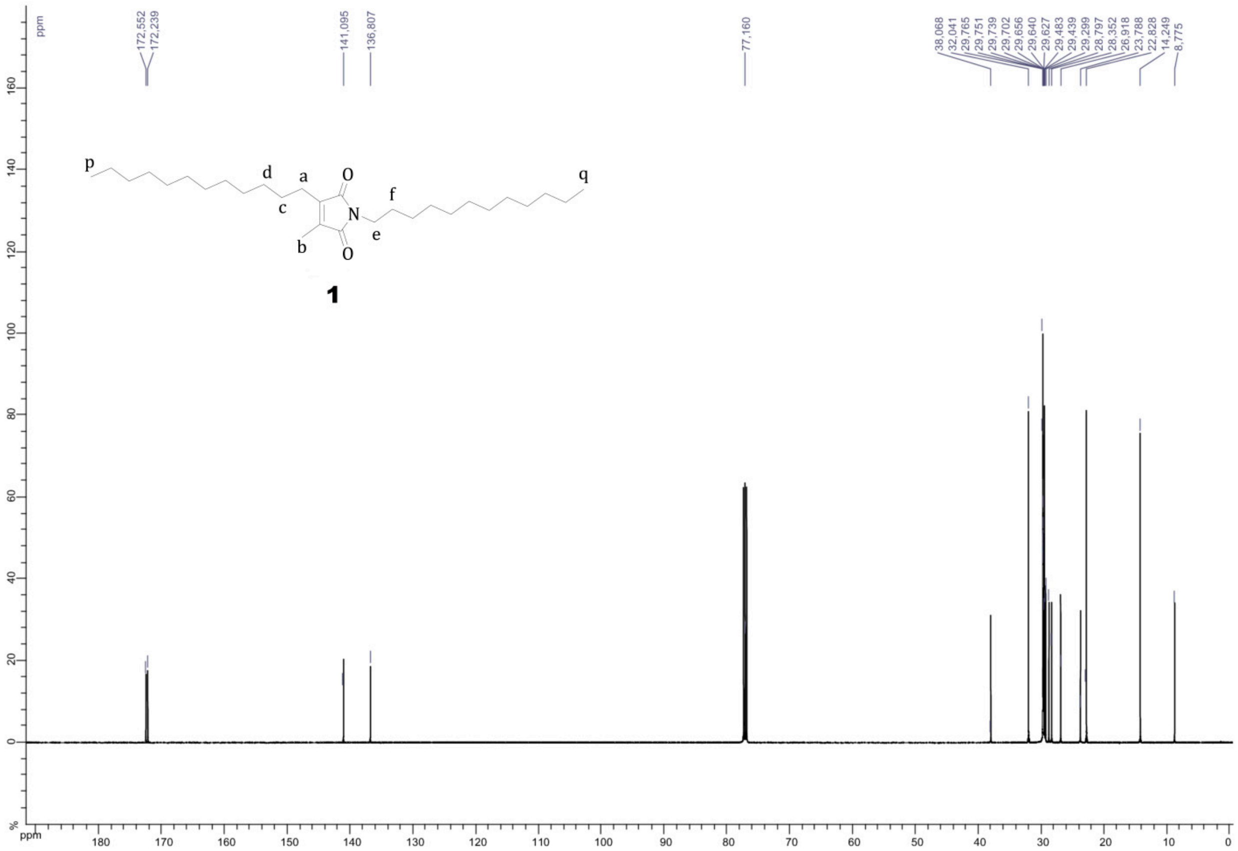

Figure S2. ^{13}C-NMR of compound **1**.

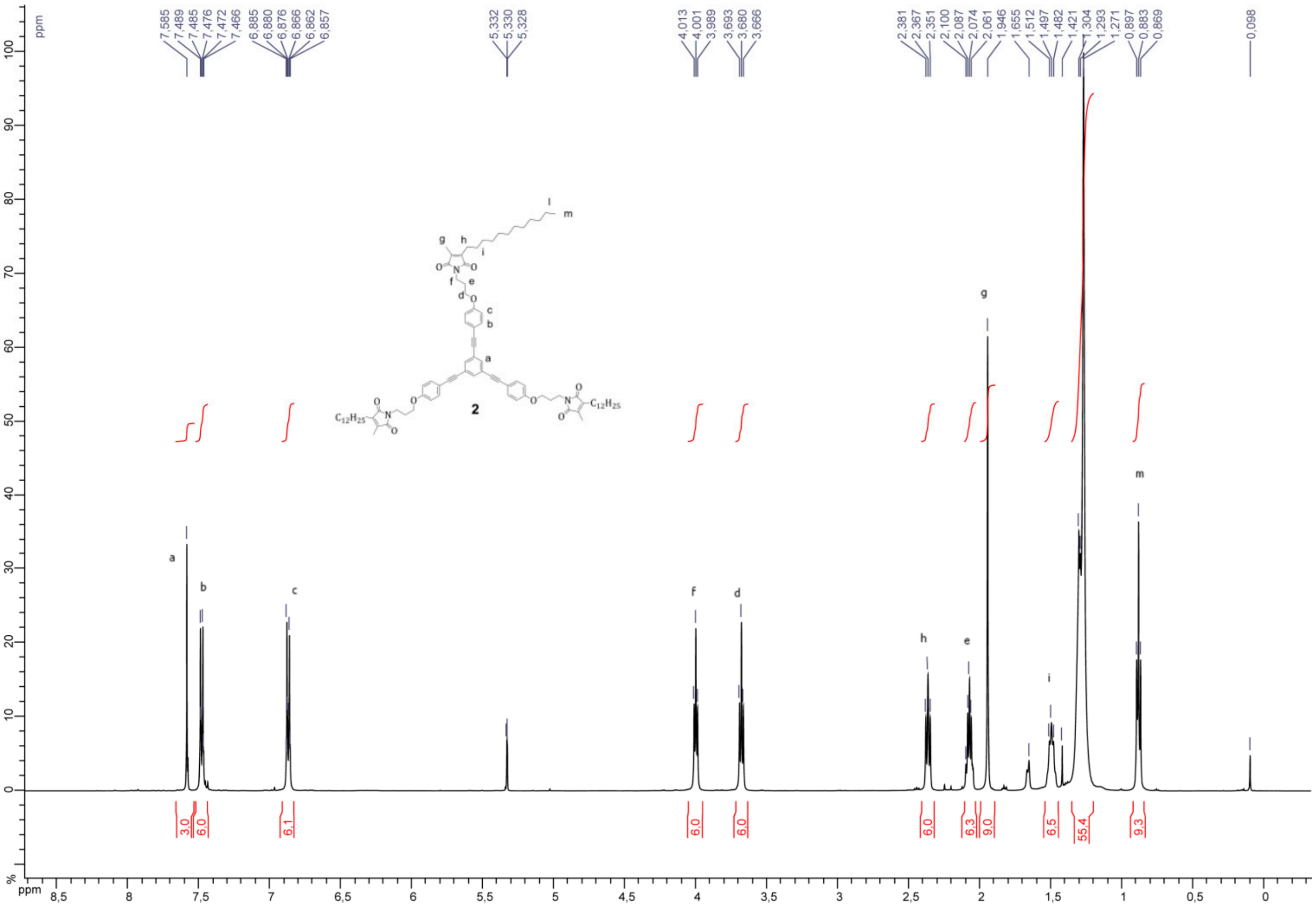

Figure S3. ^{1}H-NMR of compound **2**.

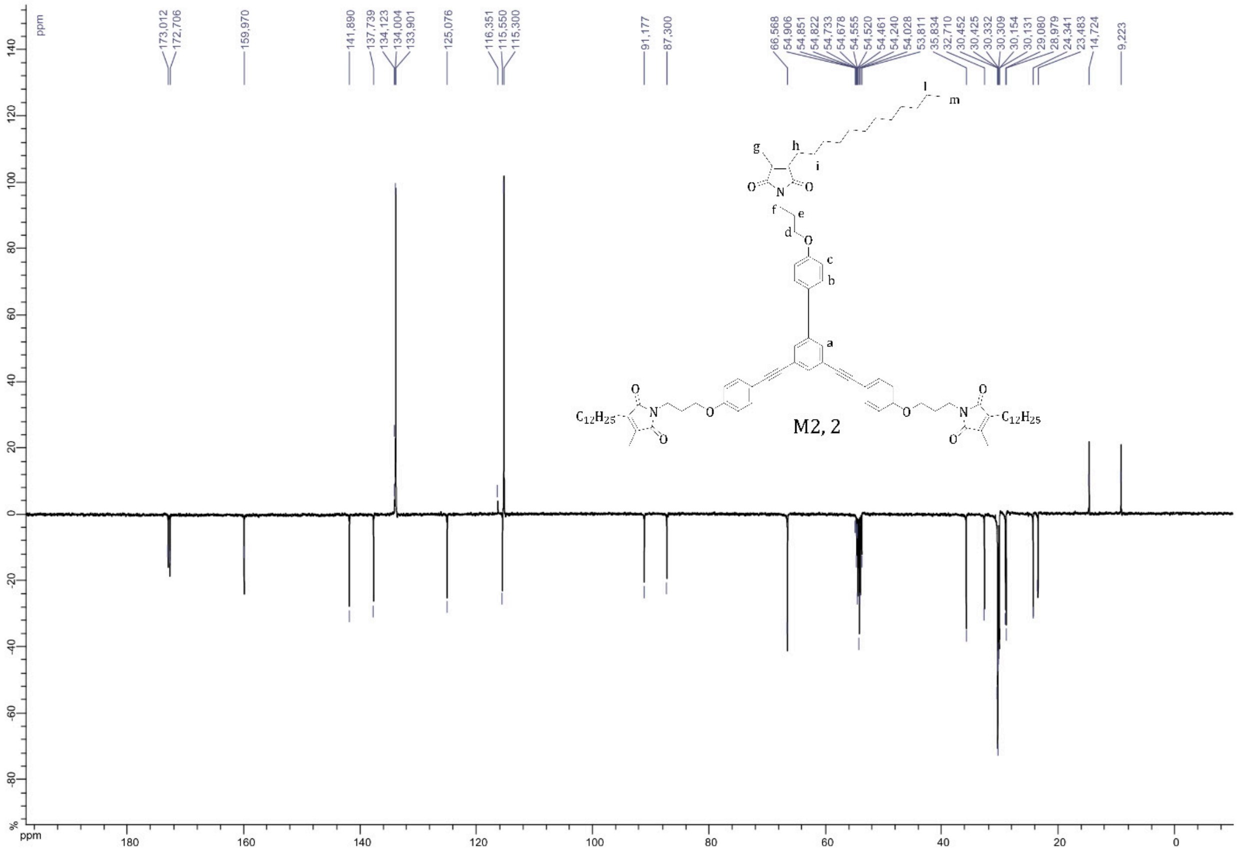

Figure S4. ^{13}C-NMR of compound **2**.

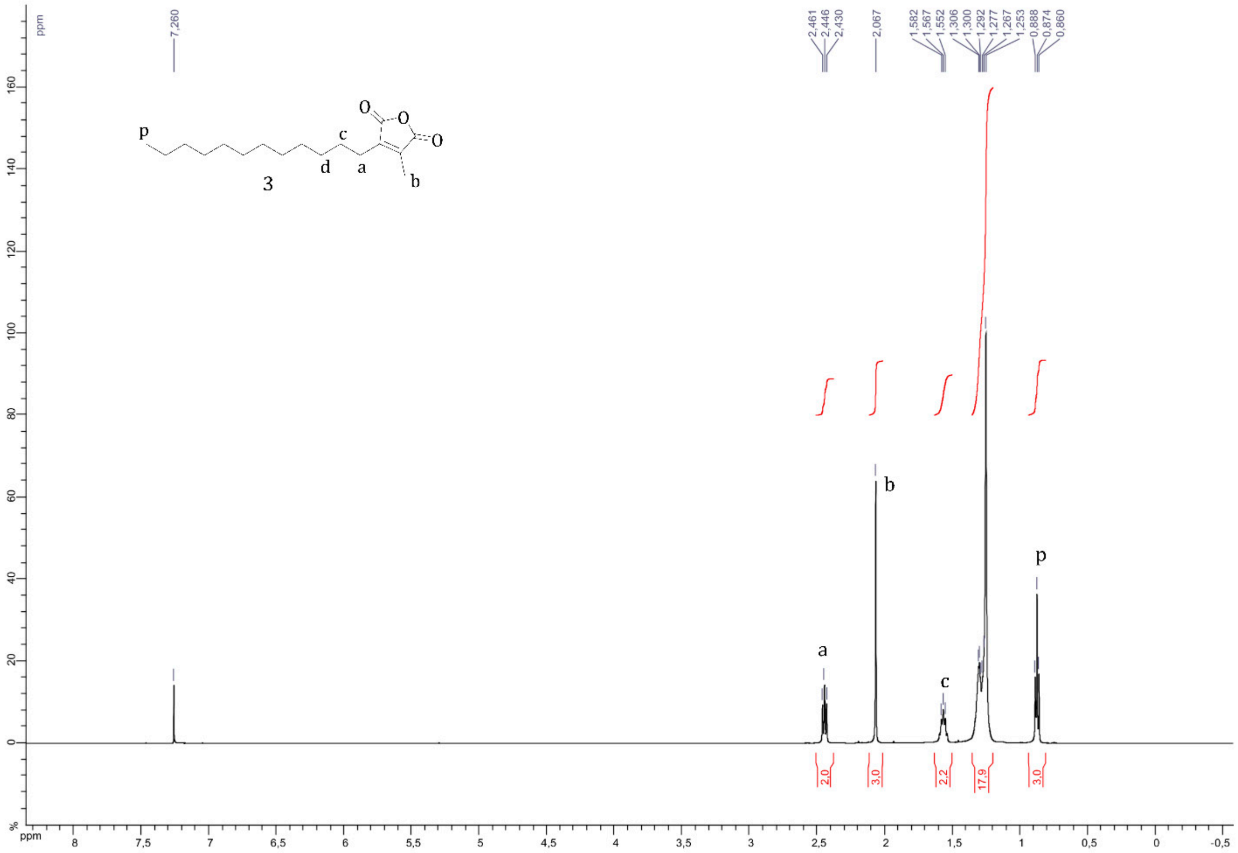

Figure S5. ^{1}H-NMR of compound **3**.

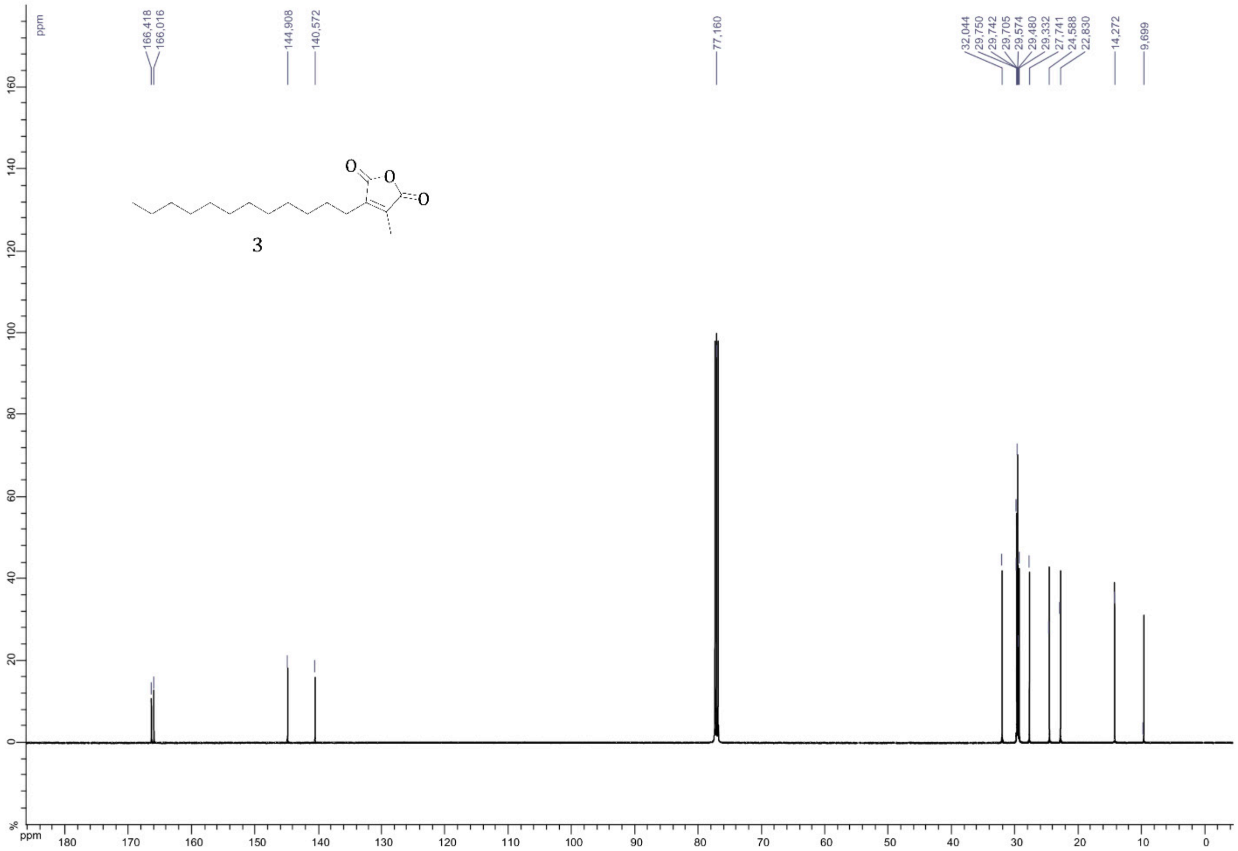

Figure S6. ^{13}C-NMR of compound **3**.

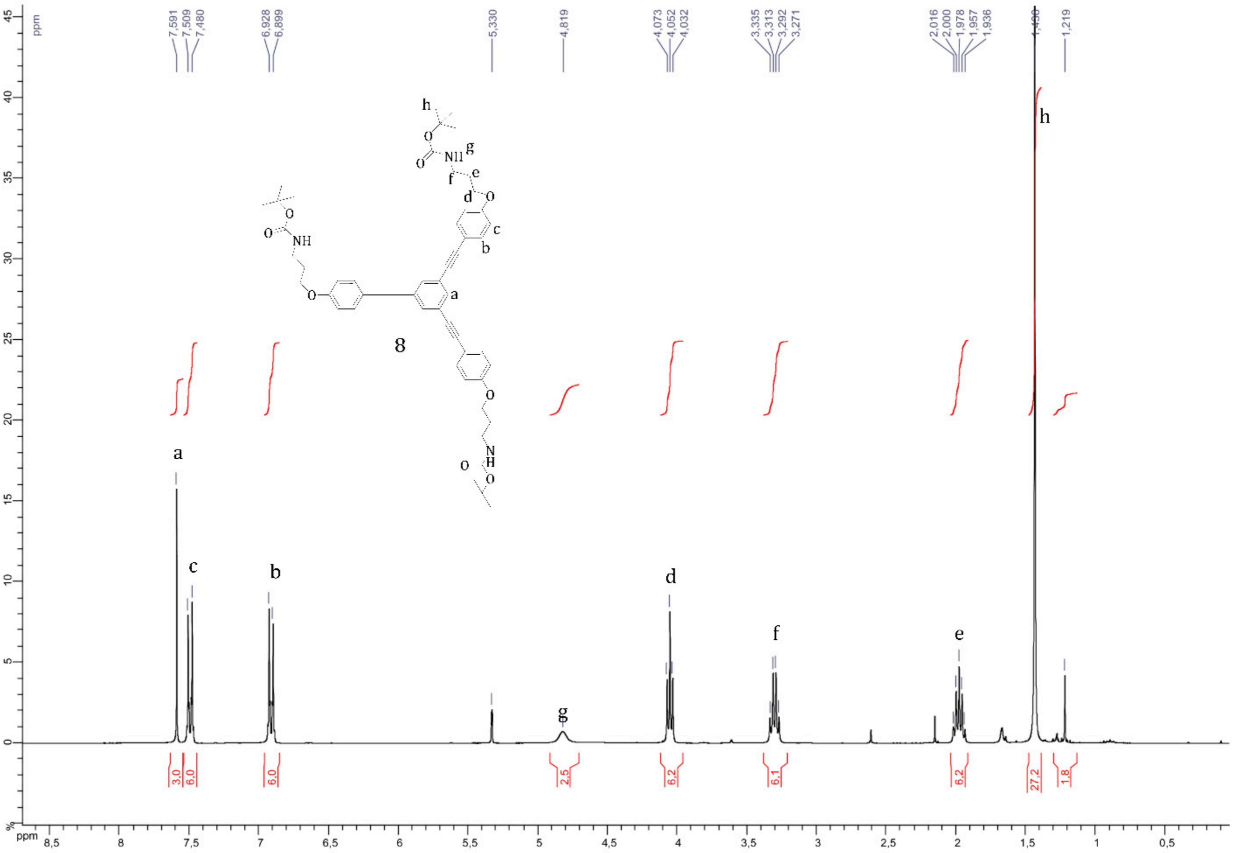

Figure S7. ^{1}H-NMR of compound **8**.

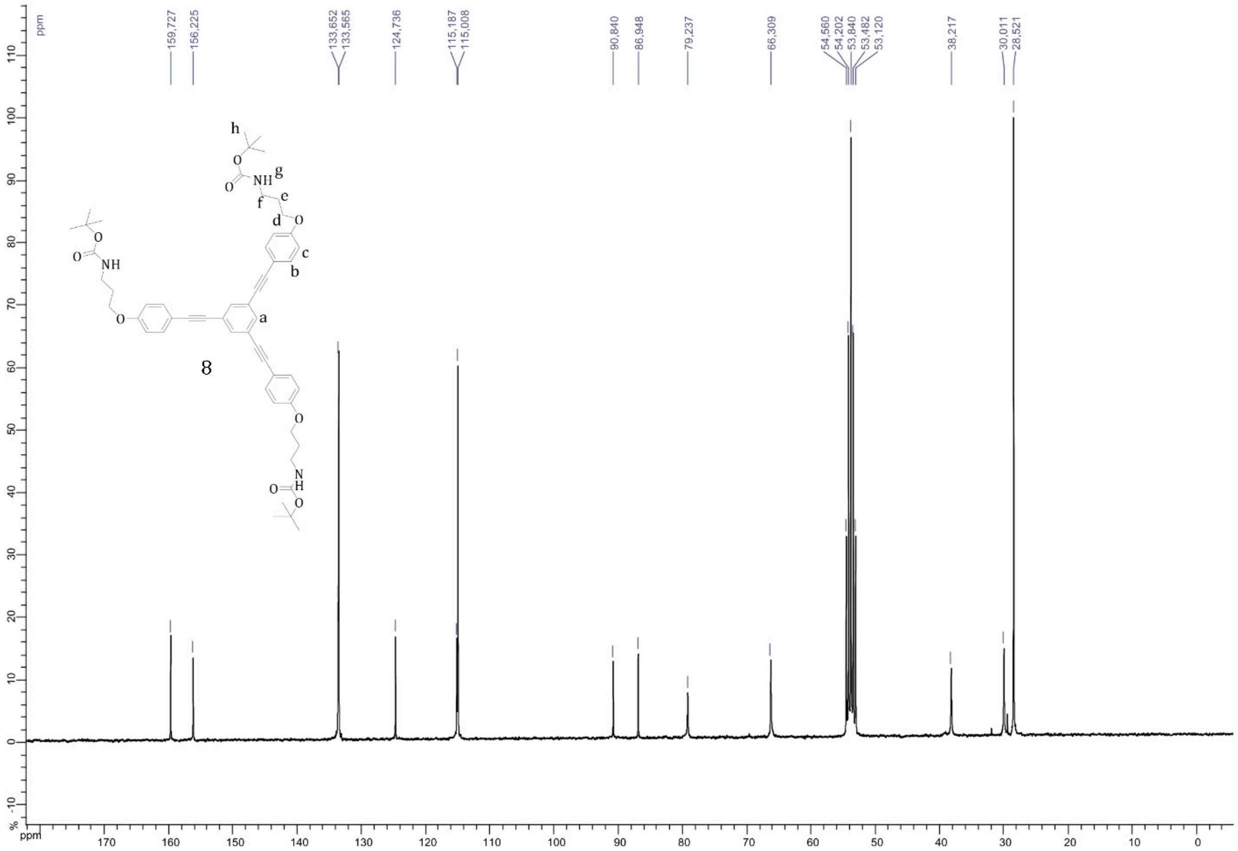

Figure S8. ^{13}C-NMR of compound **8**.

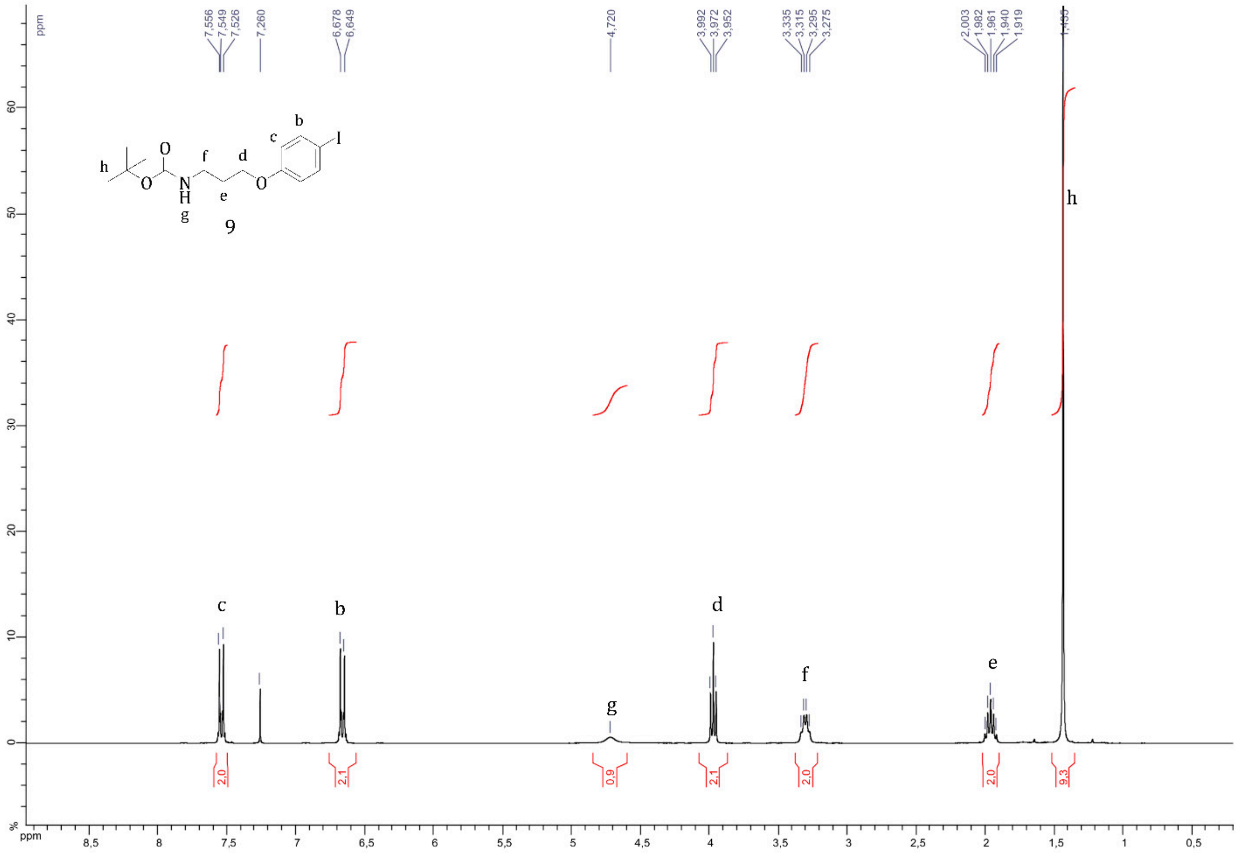

Figure S9. ^{1}H-NMR of compound **9**.

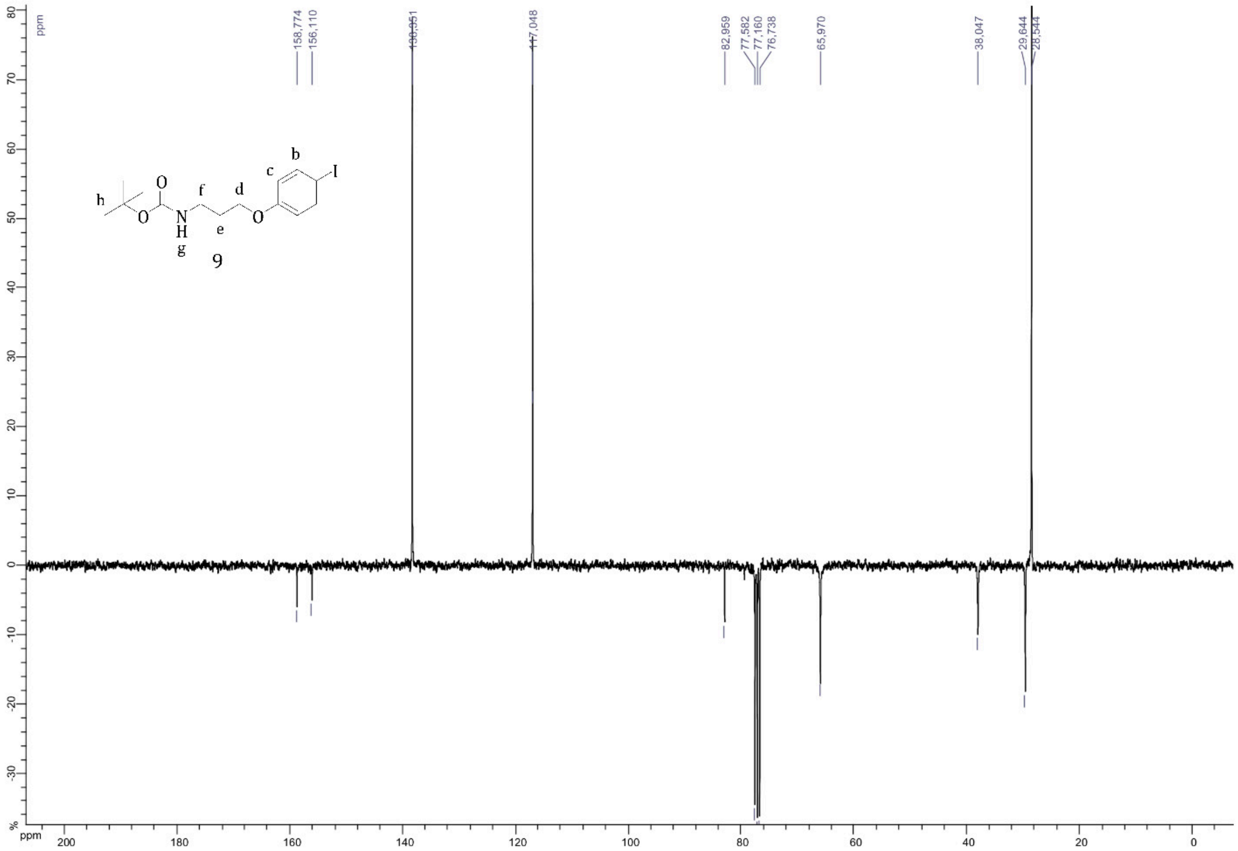

Figure S10. ^{13}C-NMR of compound **9**.
